# Do goats exhibit prosocial motivation? Insights from a novel food-giving paradigm

**DOI:** 10.1098/rsos.250556

**Published:** 2025-05-21

**Authors:** Annkatrin Pahl, Jean-Loup Rault, Jim McGetrick, Anja Eggert, Christian Nawroth, Jan Langbein

**Affiliations:** ^1^ Research Institute for Farm Animal Biology (FBN), Dummerstorf, Germany; ^2^ Institute of Animal Welfare Science, University of Veterinary Medicine, Vienna, Austria; ^3^ Department of Behavioral and Cognitive Biology, University of Vienna, Vienna, Austria

**Keywords:** altruism, cognition, Fake Apple Tree, farm animals, prosociality, ungulates

## Abstract

Research on prosociality in animals has largely focused on a few model species and a limited range of experimental paradigms. To expand this scope, we developed an ecologically relevant food-giving paradigm, the Fake Apple Tree, designed to assess prosocial motivation in goats (*Capra hircus*) by stimulating their natural climbing behaviour. In this set-up, when a ‘donor’ goat stepped onto a platform attached to a pivoting arm, the arm lowered a food dispenser within reach of conspecific ‘recipients’, while the donor itself could not access the reward. Ten out of twelve goats spontaneously learned to operate the device. In dyadic trials, goats interacted with the Fake Apple Tree more frequently when the food dispenser was active compared to control sessions where no food was provided. The frequency of interactions remained stable across test sessions. We classified platform interactions as prosocial if the donor left without approaching the food dispenser and selfish if it attempted to access the food afterwards. Consistent with findings in primates, prosocial interactions were significantly longer than selfish ones. Our results provide insights into potential prosocial tendencies in goats and highlight the utility of ecologically relevant paradigms in studying cooperative behaviours in ungulates.

## 1. Introduction

Prosociality has been historically thought to be a distinct human trait. However, over the last few decades, research has revealed that many animals show the remarkable trait of caring for others [1–5]. According to current definitions, prosocial behaviour includes a wide range of social actions that improve the well-being of others, such as helping, sharing, cooperating and comforting [6,7]. Still, we do not know how common prosocial behaviours are in the animal kingdom, making it difficult to assess its evolutionary trajectory.

The evolutionary emergence of prosociality is often explained through kin selection, in which the fitness of direct relatives, such as offspring, benefits from prosocial acts of their parents (e.g. through feeding, protection or teaching [8]). Additionally, parents may have enhanced infant survival by detecting stress signals in their offspring, which likely contributed to the evolutionary development of empathy [8]. Burkart *et al*. [9] demonstrated that in group service paradigms, which are common set-ups for testing prosociality, prosocial behaviour across species was best predicted by allomaternal care. In species providing allomaternal care, other individuals than the parents help to take care of the young. Therefore, acting prosocially towards the offspring of others within a group is likely to be advantageous in terms of one’s own fitness. Since members of groups are often related to each other, helping to care for other conspecific’s infants may enhance the survival of one’s own genes (Hamilton’s rule [10]).

Between unrelated animals, helping others may involve the expectation to receive a benefit in the future [11]. On a proximate level, social tolerance as well as empathy are discussed as a strong driver for prosocial behaviour [8,12–14]. Social tolerance facilitates cooperative and affiliative behaviours by lowering aggression and therefore allowing close proximity [13,14]. Awareness of the emotional states of other animals and the motivation to positively influence their well-being helps to promote and enhance group living [8].

Most research on prosociality has been focused on non-human primates [15], with experiments designed to match their anatomical characteristics, such as tasks requiring them to pull a bar with their hands or muzzle or to choose an object that provides a reward for a conspecific. Recently, research started to expand its interest to other groups, including birds [16,17] as well as domestic companion [18,19] and farm animals [20].

Since some of those species are not naturally inclined to pull bars, and performing it might be challenging for them, experimental set-ups need to be adjusted in order to better align with their anatomical and behavioural characteristics. Therefore, Horn *et al*. [16,21] constructed an experimental aviary designed to investigate proactive prosociality in different corvid species. The set-up included a see-saw mechanism that was activated when an individual bird landed on a perch, thereby bringing food within reach of their group mates. By taking this approach, the natural flying and landing behaviours of birds were taken into account in order to better align with the behavioural ecology of these species. Besides the ability to operate with the experimental devices used, the context of the experimental testing method can also influence an animal’s performance. For instance, dogs were shown to act prosocial towards familiar conspecifics in set-ups in which they were required to pull a bar [22] or touching tokens [23], but not in a location choice task [24]. This highlights the importance of the right methodology to test for prosociality in different species.

Several studies also employed a so-called group service paradigm, in which one animal can choose to perform an action to provide food to conspecifics without being able to access the food reward themselves (corvids [16,21]; chimpanzees [13]). Although these studies relied on the same conceptual paradigm, the protocol and design did vary substantially. Horn *et al*. [16,21] used a mechanism involving a perch on which birds could land to move a see-saw that provided food for conspecifics in an adjacent aviary. Van Leeuwen *et al*. [13] provided a button that chimpanzees could push with their hands to activate a juice fountain from which the other group members could drink. These studies demonstrate how a similar research question can be addressed by adjusting the experimental design to suit the anatomical and ecological characteristics of a given species. Adjustment of experimental designs is an important consideration, and might be particularly relevant for understudied taxa.

Within fission–fusion societies, tolerant and cooperative behaviours have been evolutionary beneficial [25]. Tightening bonds between conspecifics, for example, through affiliative behaviours, likely enhances group cohesion, which may improve collective success in predator defence or finding new food resources. For instance, feral domestic goats (*Capra hircus*) live in loose matrilineal dynamic social groups, reflecting a highly complex fission–fusion social structure [26–28]. They appear to establish particularly strong social bonds under harsh environmental conditions, which likely reflects a benefit resulting from those relationships [29]. These characertistics make ungulates, especially domestic goats, an interesting model to expand research in the field of prosocial behaviour. Since goats are being held as livestock in many countries all over the world, providing more insight into their social dynamics might also be crucial for improving their well-being. Goats are well adapted to steep mountainous terrain, which makes them proficient climbers [30]. They have been observed to forage at variable heights, including rearing onto their hind legs [31], and have been reported to climb onto trees to feed [32]. Captive goats also seem to prefer feeding at heightened positions, as feed intake was shown to increase [33,34] and agonistic interactions were shown to decrease [34] when goats were able to feed from elevated feeders. One reason for this might be improved predator detection, given that feeding from the ground restricts the field of vision [35].

For this study, we designed an apparatus to elicit prosocial motivation in goats that relies on their natural foraging and associated climbing behaviour. This apparatus—coined the Fake Apple Tree—consists of a pivoting arm to which a platform is attached on one side and a dispenser that delivers food on the other. If a goat pushes down the platform with its foot, acting as ‘donor’, the dispenser lowers down and thus comes within reach of other goats, the ‘recipient(s)’. While standing on the platform, the donor goat is not able to reach the food. One major advantage of the Fake Apple Tree is that no active training is needed for the goats to operate the device. Compared to other paradigms (e.g. [16,21–23]), no operant conditioning training had to be implemented to make the subjects interact with the experimental set-up. Similar to the group serving paradigm conducted by van Leeuwen *et al*. [13], subjects had to learn to operate the device by themselves, although the attention of the subjects was drawn towards the set-up at the beginning [13] and/or throughout each session.

Goats live in fission–fusion societies, are sociable and show excellent problem-solving abilities [36–38]. We therefore expected that they would learn to use the experimental apparatus spontaneously and to subsequently show prosocial behaviour. If the motivation to make the food available to others, and not merely exploratory behaviour, drives interactions with the apparatus, we predicted that the frequency of use of the platform to vary depending on whether a reward is available or not. If this behaviour is due to the novelty of engaging with the apparatus, we expected a decrease in the frequency of interactions with the apparatus across test sessions. We also expected to see a difference between prosocial and selfish motivation: if the goats’ motivation to interact with the platform was selfish, we expected them to approach the food dispenser in the first 5 s after leaving the platform. Contrary to that, we expected the goats to not reach for the reward after leaving the platform when they were pushing to provide food for their conspecifics. In a previous group service paradigm with chimpanzees, donors spent more time delivering food when they acted prosocial [13]. Therefore, we expected that goats would spend more time pushing down the platform if they were prosocially motivated.

## 2. Animals, material and methods

### 2.1. Subjects and housing

Two groups of female Nigerian dwarf goats each with six individuals participated in the study. Goats were approximately 1 year old, and group composition did not change for six months prior to the study. Every group included one pair of full siblings. The animals were housed in pens (4 × 3 m) littered with straw, with ad libitum access to water and hay. Concentrate was provided twice a day (150 g d^–1^ per animal on average). Stables were equipped with windows but were also provided with additional artificial light from 07.00 h to 14.00 h. To stimulate climbing behaviour and to enrich the housing conditions, a wooden pyramid with three levels was attached to the pen wall. The animals were not food restricted before testing.

### 2.2. Description of the Fake Apple Tree testing apparatus

**Figure 1.**
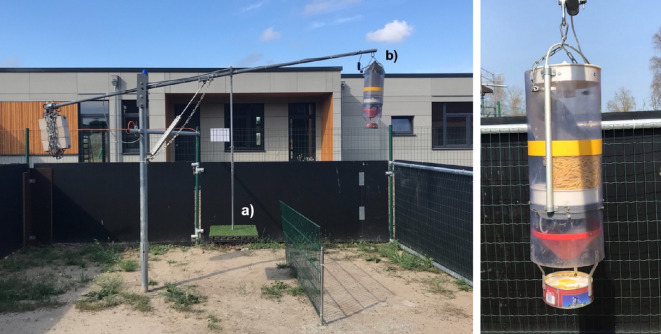
Left image: the test arena with the Fake Apple Tree apparatus on which the platform (a) and dispenser (b) were attached. Right image: the dispenser, containing raw pasta, attached to the pivoting arm.

The tests took place in two identical, adjacent outdoor arenas next to the goat facility, with wooden side walls to prevent visual distractions. The two arenas (5.5 × 7.3 m) were equipped with identical testing apparatuses that were constructed in-house. The two Fake Apple Tree testing apparatuses (figure 1, left) consisted of a pivoting arm (3.6 m) with a platform (0.5 × 0.5 m) and a dispenser (figure 1, right) containing food (raw pasta) attached to one stake. A counterweight on the opposite side of the pivoting arm kept the dispenser raised in its default position (figure 1, left). By climbing onto the platform, a goat (the ‘donor’) can lower the platform, which results in lowering the dispenser such that other individuals in the arena can gain access to the food released by the dispenser. Food was released from the dispenser every 5 min to attract the attention of the goats towards the food items. It fell into a bowl attached to the dispenser from which goats could feed if the dispenser was lowered. The upward swing of the pivoting arm after releasing the platform was softened by a pneumatic shock absorber (pneumatikatlas.de, XL 32/400 hub). A warning lamp (ComPro BL70 Warnleuchte) was installed on top of the stake of the apparatus to function as a visual cue when food was released by the dispenser. Food release as well as the visual light signal were automatically triggered by a timer via a remote signal (Hager EGN200; Hager Vertriebsgesellschaft mbH & Co. KG). A fence (1 × 2 m) was installed between the platform and the dispenser, to reduce the likelihood of the donor goat moving directly from the platform to the dispenser and eventually reaching the food reward for themselves.

The dispenser was pre-loaded before each test session. The timer was set via an app (Hager Mood v. 1.10.0) on a smartphone. Every signal induced the release of 14 g of raw pasta. The plate was big enough to hold multiple releases, in case no food was eaten and to prevent overflow of pasta onto the ground.

### 2.3. Habituation, learning and testing

#### 2.3.1. Habituation to the dispenser

Prior to testing, goats were habituated to the dispenser within their home pens inside the facility. Commercially available pellets were used in this phase as the goats were used to it, and we expected them to habituate better to the dispenser when filled with a familiar food type. The signal was given approximately 10 times a day until the group had consumed their daily portion of concentrate. This procedure was carried out because the sound of the dispenser during food delivery led to a startle response in the goats prior to habituation. The dispenser was available in one group for two to three consecutive days before providing it to the other group for the same amount of time. Goats were habituated to the dispenser over a period of three weeks.

#### 2.3.2. Habituation to the test arenas

Simultaneously, the goats were habituated as a group to the test arenas, by letting each group explore the arenas for 15 min on every weekday for three weeks. Groups alternated between the two arenas. The testing apparatus was already installed in the test arena, but the dispensers were not attached to it. The goats were physically able to climb onto the platform but were too light to push it down, as the dispenser was needed to balance the counterweight attached to the pivoting arm.

#### 2.3.3. Group phase

A subsequent learning phase of four weeks was implemented to provide goats with the opportunity to learn the contingencies of the test apparatus autonomously. Both groups were led into the arena separately and subjected to learning sessions performed daily on weekdays for 2 h each. Food was released by the dispenser every 15 min.

At first, goats had the opportunity to spontaneously interact with the platform. If no goat jumped onto the platform, the dispenser and platform were fixed to the ground for the whole duration of the next session, providing the goats with access to the food. This was intended to reinforce the association between the dispenser and the food. In the next session, the platform and dispenser were in the default raised position again. The goats that had already interacted with the platform at least once were removed for the next session to avoid monopolisation of the platform by experienced goats and to allow other group members to interact with the platform. A maximum of four goats were removed to allow the remaining two goats to interact with each other via the apparatus. All goats of group 1 and four goats from group 2 pushed the platform down on their own at least once after a period of four weeks and various numbers of training sessions. The two subjects that did not interact with the platform remained in group 2 and also participated in both of the following testing phases. The goats were provided four more weeks of learning in a group setting, in order to provide them with opportunities to gain more experience with the apparatus.

#### 2.3.4. Dyadic testing phase

In the test phases, all subjects in a group were paired and tested with each other, resulting in every goat of a group getting to be tested with every other subject of its group once within each test phase. Each dyad was tested three times in total over three weeks, including two test sessions and one control session. This procedure resulted in a total of 45 dyadic sessions per group. The order of sessions within each group was pseudorandomized. The respective dyad was separated from the group in their home pen immediately before the test started and gently guided into the arena. Immediately after the two goats entered the arena, the first batch of food was released. Food release was associated with a visual (warning lamp) and an acoustic (noise of the engine of the dispenser) cue. Once the first batch of food was released, testing began. Each test session lasted for 30 min. Food was automatically released every 5 min. A total of six dyads were tested per day (three dyads per group), with dyads of one group in the morning and dyads of the other group in the afternoon. In the test sessions, the dispenser was filled before goats were brought to the arena, and the food reward was dispensed as described above. In the control session, the dispenser was emptied before the test started; therefore, no food could be released, and accordingly, no visual or acoustic cues were emitted.

### 2.4. Behavioural observation

**Figure 2.**
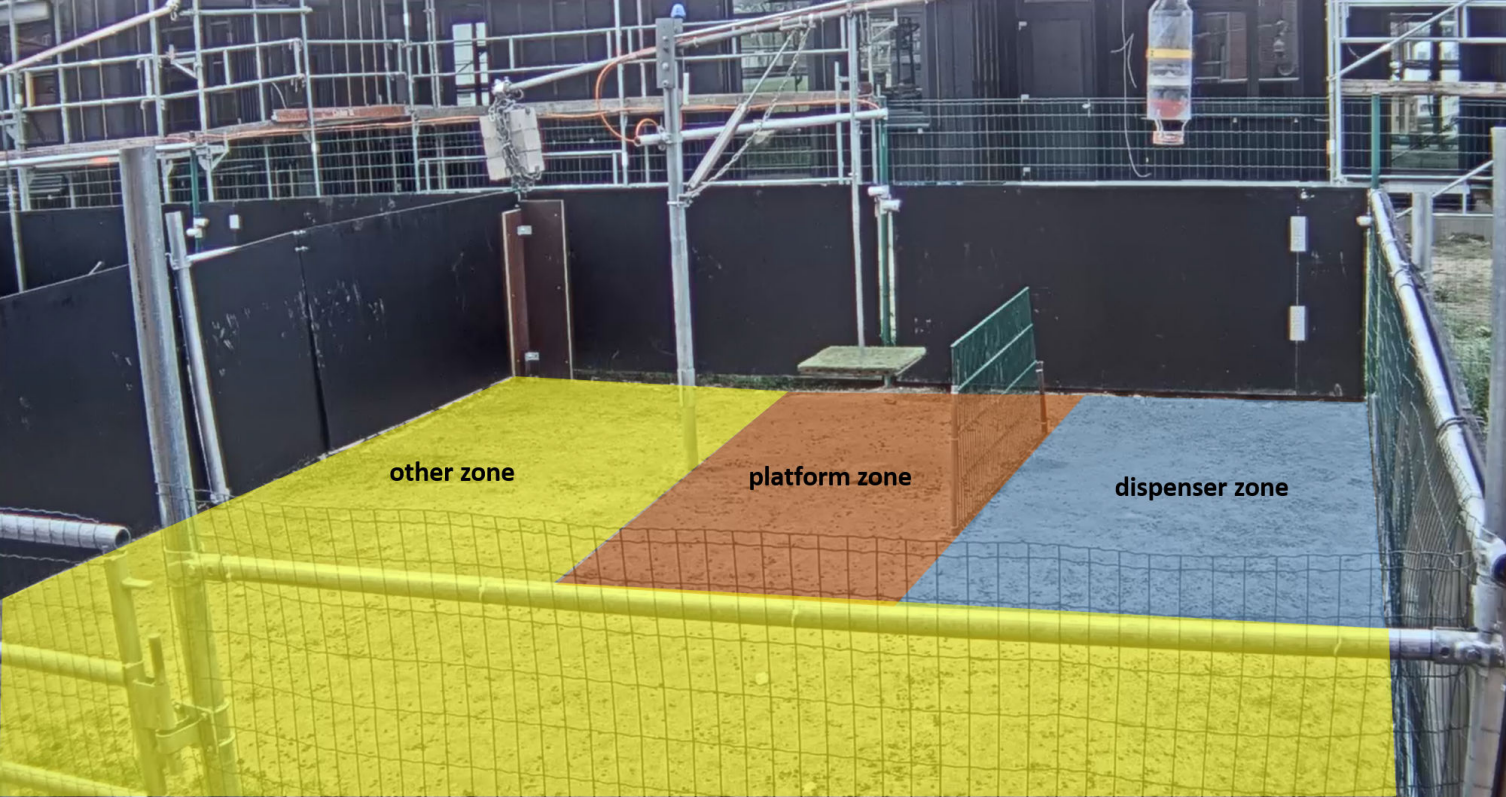
The test arena with the testing apparatus and the three different zones for observation: platform zone coloured in red, dispenser zone coloured in blue and other zone in yellow.

All sessions were videotaped (AXIS M1124; 1280 × 720 px, 15fps H.264) and processed via AXIS software (AXIS Camera Station, Client-v. 5.37.304; Axis Communications AB). Video coding was performed using the software The Observer XT v. 13.0 (Noldus IT, Wageningen, The Netherlands). The behaviour of each dyad was coded regarding the goats’ interactions with the platform. An interaction is defined as follows: a goat touches the platform with at least both front hooves and thereby pushes it down. Coding started as soon as the goat put both front hooves on the platform. The interaction lasted until the goat no longer touched the platform with any hoof. We coded both the frequency and duration of platform interactions for each subject as well as the subject’s location 5 s after it had left the platform. We chose this value as a threshold as we observed that this was the approximate maximum time that a goat needed to enter the dispenser zone from the platform when moving in a straight line. In order to track the location of the goats, the test arena was divided into the following three zones: a ‘platform zone’, a ‘dispenser zone’ and an ‘other zone’ (figure 2). platform interactions that were followed by an attempt by the donor animal to reach the reward for itself (i.e. by entering the dispenser zone within 5 s after having left the platform) were categorized as ‘selfish’. Pushing events that did not entail such an attempt were labelled as ‘prosocial’. The software Golden Ratio v. 3.1 was used to visually divide the arena for observation. For alignment, we used the fence that separated the platform from the dispenser and the rod of the testing apparatus as reference points. From the ends of the fence, zones stretched 1 m to the front and rear, respectively. The various zones differed in size but were chosen based on their functional relevance.

### 2.5. Social relationships

To test whether dominance hierarchy and sociality had an influence on prosocial motivation, a food competition test [39,40] as well as a scan sampling of laying behaviour in the home pen [41] was conducted prior to the main experiment. For the dominance hierarchy, food concentrate was offered to the goats, right before the observation started. Both groups were tested individually. For 15 min, the following interactions were verbally recorded on a voice recorder: head butting (striking with head on other goats), threatening (moving the head quickly towards another goat), chasing (quick moves towards retreating goats) and displacement (pushing other goats which gave way as a result). Only interactions with a clear winner and loser were included. Observations took place once a day for four weeks (Monday–Friday). For every group, data were transformed into a winner–loser table sorted by date and processed with the package ‘EloRating’ [42] for the software R Studio [43].

To measure the general sociality of every individual, over five weeks, the home pens of both groups were video recorded (AXIS M1124; 1280 × 720 px, 15fps H.264) twice a day for 70 min per session. Using the scan sampling method with the software The Observer XT by Noldus (v. 13.0), laying behaviour was recorded every 5 min. We observed whether the individuals were lying in body contact with a conspecific. A sociality index was calculated as described by Silk *et al*. [41].

### 2.6. Statistical analysis

The dataset consisted of repeated measures of 10 individual subjects interacting with different partners. The response variable, motivation, was a binary outcome (‘selfish’ or ‘prosocial’). Predictor variables included subject ID, session type (control, test 1, test 2) and the Elo rating of the partner as a measure of the animal’s dominance. Each subject interacted with three to five partners across the different sessions.

Data were analysed using the R statistical software (v. 4.2.2) [43]. Given the repeated measures structure, a binomial generalized linear mixed model (GLMM) was fitted using the R package ‘glmmTMB’ to analyse the probability of motivation (binomial response: selfish/prosocial) as a function of ‘subject identity’ (10 animals) and ‘session number’ (control, test 1, test 2) and two covariates ‘dyadic sociality index’ (values ranging between 0 and 8.4) and ‘partner’s Elo rating’ (values ranging between 601 and 1730). We *z*-transformed both covariates to facilitate the interpretation of the results. Subject and session were included as fixed effects, while the subject:partner interaction was modelled as a random effect to account for repeated observations within subject–partner pairs. We assessed model performance and checked for overdispersion using the ‘DHARMa’ package, multicollinearity and inspected the residual diagnostics via ‘DHARMa’ simulated residual plots. We assessed the significance of fixed effects and covariates using type II Wald chi-square tests, as implemented in the R package ‘car’. Estimated marginal means (EMMs) were computed using the R package ‘emmeans’ to obtain subject-specific motivation probabilities ranging between 0 and 1. Probabilities <0.5 can be regarded as a prosocial motivation, while values >0.5 can be regarded as a selfish motivation.

Linear mixed models (LMM) were used to analyse the frequency of platform interactions, using the R package ‘lmerTest’ [44]. The response variable was the total number of platform interactions per individual per session, with ‘session’ (three levels: test 1, test 2, control) included as a fixed factor. To fit all model requirements, data of the response variable were log-transformed. Partner identity and mother of subjects nested within individual identity were included as random factors. We used the ‘Tukey’ post hoc test for post hoc comparisons using the packages ‘emmeans’ and ‘multcomp’ [45].

We used an LMM to determine whether durations of platform interactions statistically differed between what we defined as ‘selfish’ and ‘prosocial’ pushing events. The data were log-transformed to fit model requirements. As a fixed factor, we set the variable ‘motivation’ (two levels: prosocial, selfish), whereby ‘prosocial’ was defined as platform interaction after which the subject did not enter the dispenser zone within 5 s after leaving the platform, whereas ‘selfish’ was defined as a platform interaction after which the subject entered the dispenser zone within 5 s after leaving the platform. Again, we included partner identity as well as the mother of subjects nested in individual identity as random factors. The alpha level was set at 0.05.

## 3. Results

From the first group learning session onwards, goats spontaneously interacted with the platform without any additional training administered by humans. By the end of the group learning phase, 10 out of 12 goats had successfully interacted with the platform by pushing it down.

**Figure 3.**
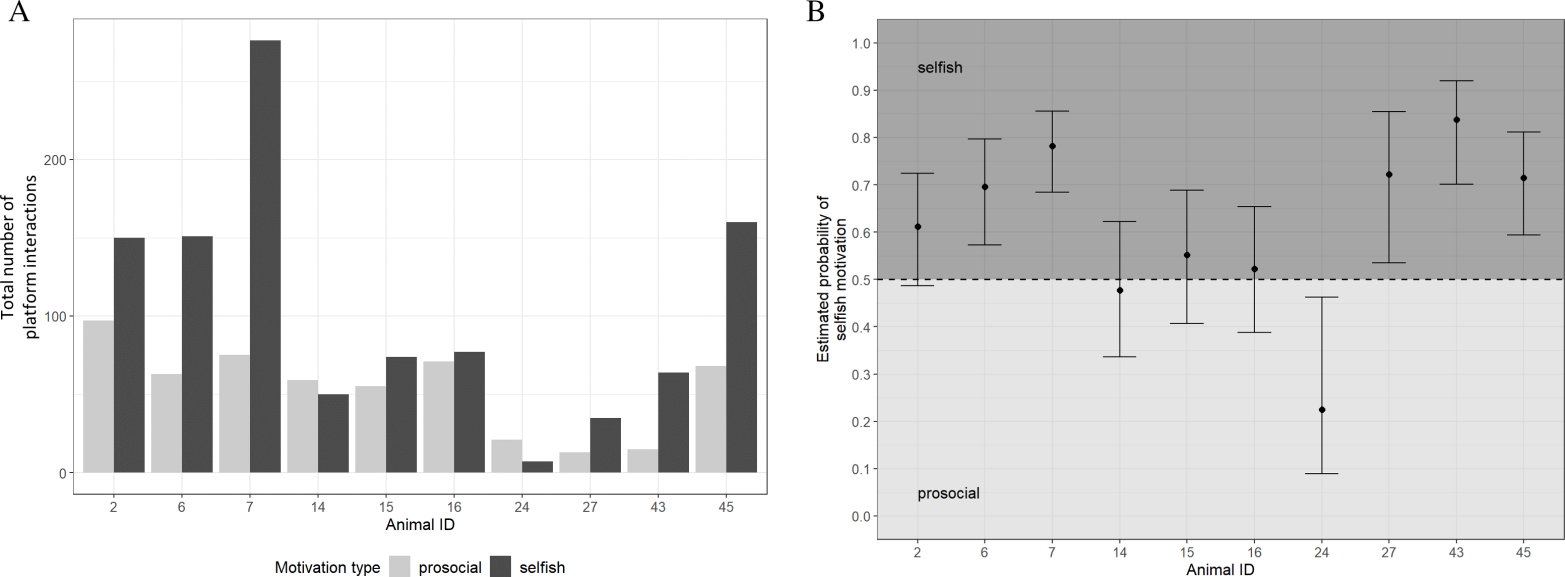
(A) Total number of platform interactions of 10 individuals across all three sessions, divided into ‘selfish’ and ‘prosocial’ interactions. (B) Estimated probability of selfish motivation. Animals with values <0.5 are regarded as prosocial animals, and animals with values >0.5 are regarded as selfish animals. Presented are EMMs with 95% confidence intervals of a binomial GLMM.

In all three sessions, individuals pushed the platform between 28 and 351 times in total (figure 3). Based on the donor’s location 5 s after leaving the platform, no subject was interacting with the platform exclusively ‘selfish’ or ‘prosocial’. In total, 66% of interactions were followed by an attempt of the donor animals to reach the food reward themselves (1044 selfish interactions, 537 prosocial) but with inter-individual variation (figure 3). The binomial logistic regression model revealed that both subject identity and session had a significant effect on motivation (subject: chi-square = 37.3, *p* < 0.001; session: chi-square = 45.7, *p* < 0.001). In contrast, neither the DSI nor the Elo rating of the partner significantly influenced motivation (chi-square = 0.50, *p* = 0.478; chi-square = 1.26, *p* = 0.262).

The EMMs indicated that selfish interactions were significantly less frequent in the control session, when the dispenser was empty (probability = 0.469 (0.382; 0.558)), compared to the test sessions where food was available (test 1: 0.586 (0.527; 0.643); test 2: 0.737 (0.680; 0.787)). This suggests that, on average, goats exhibited more selfish motivation when the dispenser contained food. However, significant differences among subjects indicate substantial between-individual variability across all three sessions. In the control session, 5 out of 10 goats displayed more selfish motivation, while the other 5 showed more prosocial motivation. In test 1, 6 out of 10 goats were more selfishly motivated, and in test 2, this increased to 9 out of 10 goats.

EMMs averaged over the three sessions show that six of the animals showed selfish motivation. Only animal 24, with an estimated probablity of 0.220 (0.085; 0.463), can be regarded as being prosocially motivated. Animals 14, 15 and 16 showed ambiguous behaviour, oscillating between selfish and prosocial motivations, and the remaining six animals showed a clear selfish motivation (figure 3B).

**Figure 4.**
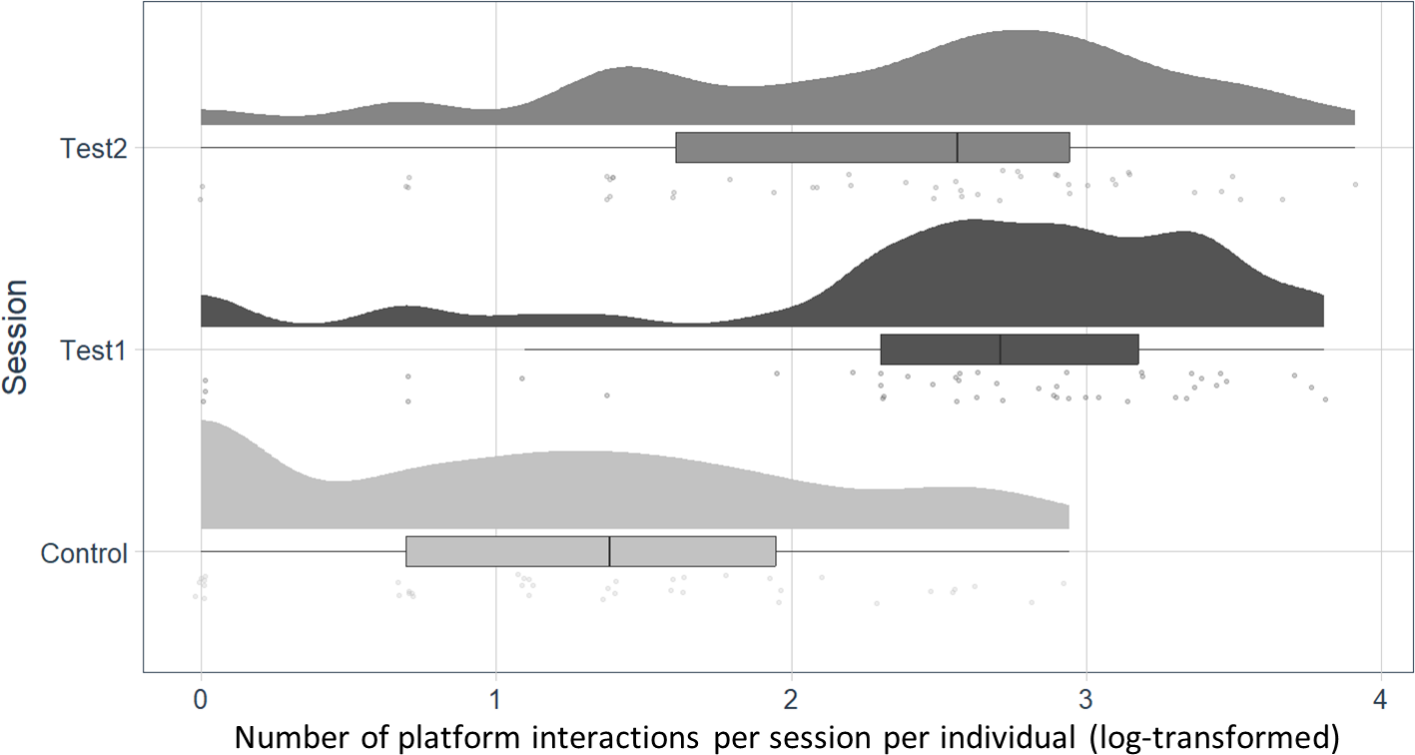
Number of platform interactions per session per individual (log-transformed) in all three sessions (control, test 1, test 2). Boxes show the interquartile range (25th percentile to 75th percentile) and median. The variability outside the upper and lower quartiles (up to 1.5 times the interquartile range) is depicted by the whiskers. Points represent the sum of platform interactions of an individual per session.

Goats interacted more often with the platform in the test sessions compared to the control session (figure 4): test 1 versus control: *p* < 0.001, *z* = 7.678; test 2 versus control: *p* < 0.001, *z* = 6.065. In contrast, we found no difference in the frequency of platform interactions between test 1 and test 2 (*p* = 0.197, *z* = −1.720).

**Figure 5.**
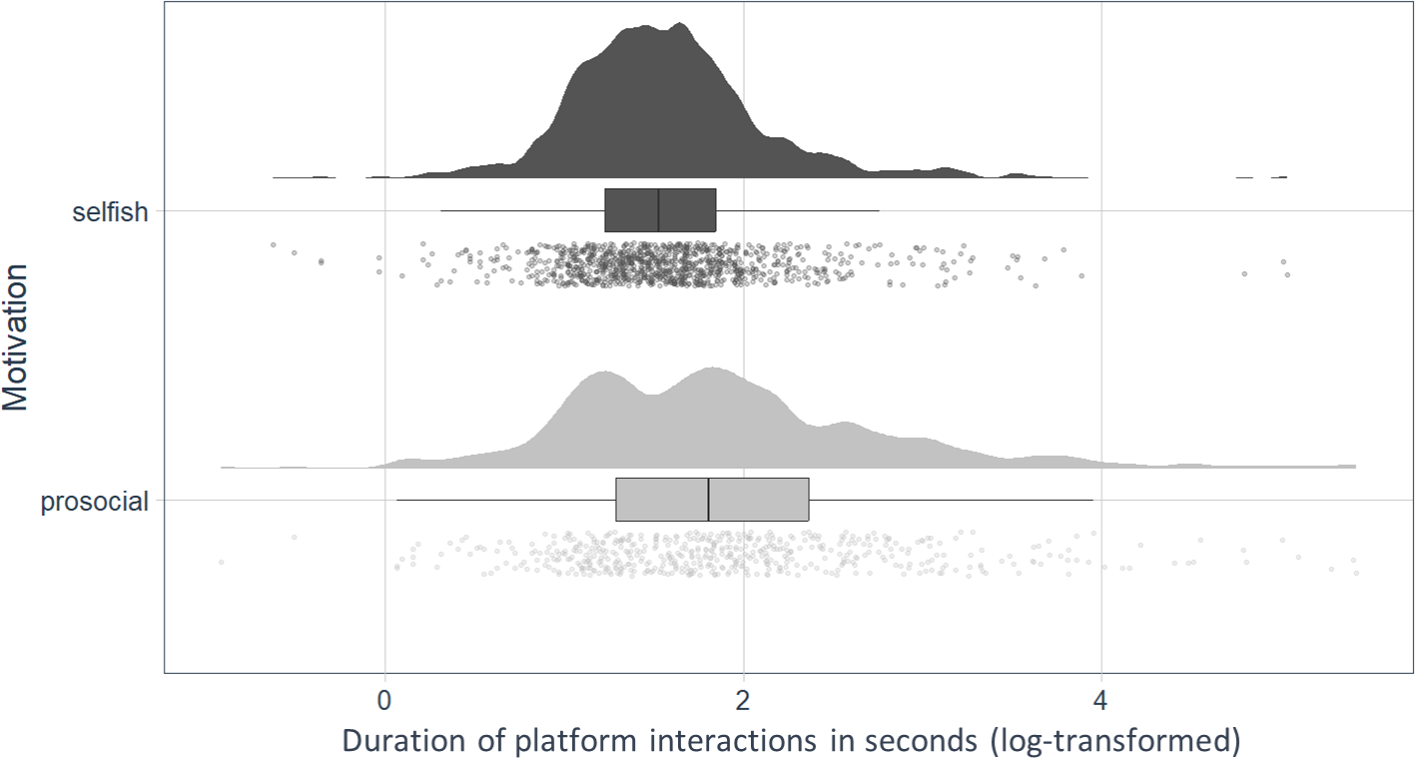
Duration (log-transformed) of platform interactions, divided into selfish and prosocial acts. Boxes show the interquartile range (25th percentile to 75th percentile) and median. The variability outside the upper and lower quartiles (up to 1.5 times the interquartile range) is depicted by the whiskers. Points represent the duration of individual platform interactions.

Instances in which goats entered the dispenser zone 5 s after leaving the platform (selfish) were preceded by shorter durations on the platform compared with instances in which they did not enter the dispenser zone after leaving the platform (prosocial; *p* < 0.001; *df* = 1; *F* = 35.228; figure 5).

## 4. Discussion

We developed a novel food-giving paradigm to test for behaviour that could be interpreted as prosocial motivation in dwarf goats, with the ungulate taxa being underrepresented in research on prosociality in animals. Goats were able to spontaneously learn to operate the apparatus that provides access to food for conspecifics when a donor animal climbs onto a platform attached to the apparatus. In dyadic test situations, goats interacted with the platform more frequently in test sessions when the dispenser contained food that could be made accessible to conspecifics, compared to the control session in which the dispenser was empty. This suggests that goats were able to distinguish between the presence versus absence of food and that food delivery to the recipient goat could play a role in the motivation to engage with the device. Goats showed a high level of interactions with the platform that did not appear to entail a selfish motivation to gain access to the food for themselves. Those interactions were also of longer duration compared to platform interactions after which the donor goat showed an attempt to approach the dispenser. This food-giving paradigm offers a promising approach to further investigate aspects influencing prosocial motivation, such as familiarity, affiliative relationship or hierarchy, in goats [20].

Goats’ successful use of this novel apparatus may relate to its ecological relevance. As browsers, goats are dependent on constantly finding new food sources such as bushes, shrubs and trees. They are adapted to steep elevations in mountainous terrains, and, therefore, climbing is part of their explorative behaviour. In the test phase, we found that goats interacted more often with the platform when food was available (test conditions) compared to when no food was available (control condition). Our results suggest that the motivation to manipulate the platform was partly, but not exclusively, motivated by the availability of food. Further research is required to disentangle whether donor goats decreased their interactions with the apparatus in the control condition because the partner could not gain access to food (prosocial motivation) or because the donor goat would itself not receive food (selfish motivation). Apart from selfish or prosocial motivations, goats could have other motivations for engaging with the apparatus, such as exploration or play behaviour (e.g. climbing), which requires further tests with other control conditions to rule out alternative explanations.

In this regard, our removal of the visual and acoustic cues that indicated the imminent food reward during the control sessions might also have influenced the goats’ engagement with the platform. While the removal of these cues was intended to clarify the absence of food to the goats, it might have reduced the goat’s motivation to interact with the platform, independent of whether they could reach a food reward or not. Future studies should therefore keep their signalling consistent in order to better disentangle the impact of food availability.

The different distributions of overall motivations (with six goats acting significantly more selfish, one goat acting significantly more prosocial and three goats acting in a balanced way) could potentially be explained through the optimal foraging theory [46]. Foraging is costly, and therefore selfish food intake may be the best way to secure one’s own fitness, particularly when it is uncertain whether the rewarded counterpart will reciprocate the prosocial act afterwards. It would be interesting to know whether the ratio of selfish and prosocial platform interactions would change in a set-up in which goats could choose between either rewarding only themselves or themselves and a conspecific, as it was already conducted in various prosocial choice tasks with other species, indicating a preference for rewarding both, donor and recipient over only the donor [47–49]. Interestingly, rhesus macaques appear to show prosocial tendencies in a prosocial choice task but almost stopped acting prosocially when only the other recipient was going to get a reward instead of both, donor and recipient [50]. Also, the same study showed that dominant individuals were more prone to act prosocial. Our results could not show any influence of dominance hierarchy on the motivation to operate the platform.

The observed distinctions in the goat’s motivation to interact with the platform raise the question of whether we can observe differences in the goat’s behaviour during seemingly selfishly motivated platform interactions compared to prosocially motivated interactions. A selfishly motivated goat would be expected to approach the food dispenser immediately after leaving the platform. However, our findings indicate that after longer platform interactions, goats were less likely to do so. This suggests that our classification of ‘selfish’ and ‘prosocial’ platform interactions is predicted by their duration. To provide food for conspecifics, platform interactions of the donor need to be long enough to allow the recipient to approach the dispenser and feed from it. It is possible that the prolonged duration of the prosocial-defined platform interactions can be considered being goal-directed, given that one criterion for goal-directedness is that the behaviour persists until the goal is met (i.e. food is consumed by the recipient) [51]. Still, mechanisms such as goal-directedness should be further investigated, as we cannot disentangle the motivations based on the present results.

Even though other research often found that dominance hierarchy (rats [52]; primates [50,53]; birds [54]) and social bond (primates [5,6]) between donor and recipient influence the display of prosociality, we did not find any correlation. It is possible that social parameters do not have an impact on the occurrence of prosocial motivation in goats. However, it is likely that the small sample size of just six goats per group was not sufficient to capture a possible effect.

We faced one major limitation in our current experimental set-up, namely the ability of animals to cheat the system. In some cases (12.9%), the donor goats were able to reach the food reward for themselves, as the dispenser returned relatively slowly to the upper, default, position. This was either because the recipient goat was able to hold the dispenser down with its snout, even though the donor goat had left the platform, or because the donor goat was particularly large and was able to pull the upward-moving dispenser down with its front legs when it reared up on its hind legs before the dispenser had returned fully to its default position. Future adaptations of the experimental testing apparatus should seek to avoid inadvertently rewarding donor animals when aiming to test for prosocial motivation. A longer fence between the platform and the dispenser, or even a complete separation between the donor and the recipient, may help to prevent self-rewarding behaviour in the future. However, the latter would also inhibit potential reciprocal behaviour within the same session [7] and could be a source of separation stress for social animals. Prosocial motivation can involve empathising with the suffering or needs of others, and stress can dampen this response [55].

## 5. Conclusion

Our subjects spontaneously learned to use an apparatus that has been designed to be ecologically relevant for goats, to provide food to their conspecifics. A modified version of this apparatus could help to assess prosocial intentions in other ungulates as well. This might give more insights into the influence of ecological parameters on the development of prosocial motivation and the evolutionary pathways of its emergence.

## Data Availability

Data and supporting information can be found here:[57].
